# Tandem‐Mass‐Tag Based Proteomic Analysis Facilitates Analyzing Critical Factors of Porous Silicon Nanoparticles in Determining Their Biological Responses under Diseased Condition

**DOI:** 10.1002/advs.202001129

**Published:** 2020-06-23

**Authors:** Yunzhan Li, Zehua Liu, Li Li, Wenhua Lian, Yaohui He, Elbadry Khalil, Ermei Mäkilä, Wenzhong Zhang, Giulia Torrieri, Xueyan Liu, Jingyi Su, Yuanming Xiu, Flavia Fontana, Jarno Salonen, Jouni Hirvonen, Wen Liu, Hongbo Zhang, Hélder A. Santos, Xianming Deng

**Affiliations:** ^1^ State Key Laboratory of Cellular Stress Biology Innovation Center for Cell Signaling Network School of Life Sciences Xiamen University Fujian 361101 China; ^2^ State‐Province Joint Engineering Laboratory of Targeted Drugs from Natural Products School of Life Sciences Xiamen University Fujian 361101 China; ^3^ Drug Research program Division of Pharmaceutical Chemistry and Technology Drug Research Program Faculty of Pharmacy University of Helsinki Helsinki FI‐00014 Finland; ^4^ School of Pharmaceutical Sciences Xiamen University Fujian 361101 China; ^5^ Laboratory of Industrial Physics Department of Physics University of Turku Turku FI‐20014 Finland; ^6^ Department of Chemistry University of Helsinki Helsinki FI‐00014 Finland; ^7^ Pharmaceutical Sciences Laboratory and Turku Bioscience Centre Abo Akademi University Turku FI‐20520 Finland; ^8^ Helsinki Institute of Life Science (HiLIFE) University of Helsinki Helsinki FI‐00014 Finland

**Keywords:** acute liver inflammation, immunogenicity, porous silicon, protein corona, R‐language

## Abstract

The analysis of nanoparticles’ biocompatibility and immunogenicity is mostly performed under a healthy condition. However, more clinically relevant evaluation conducted under pathological condition is less known. Here, the immunogenicity and bio–nano interactions of porous silicon nanoparticles (PSi NPs) are evaluated in an acute liver inflammation mice model. Interestingly, a new mechanism in which PSi NPs can remit the hepatocellular damage and inflammation activation in a surface dependent manner through protein corona formation, which perturbs the inflammation by capturing the pro‐inflammatory signaling proteins that are inordinately excreted or exposed under pathological condition, is found. This signal sequestration further attenuates the nuclear factor *κ*B pathway activation and cytokines production from macrophages. Hence, the study proposes a potential mechanism for elucidating the altered immunogenicity of nanomaterials under pathological conditions, which might further offer insights to establish harmonized standards for assessing the biosafety of biomaterials in a disease‐specific or personalized manner.

## Graphical abstract

The inflammation attenuation capability of porous silicon nanoparticles (PSi NPs) under an acute liver inflammation condition is positively correlated with the protein corona formation process. The detailed composition of protein corona composition of each type of PSi NPs is identified and confirmed by both proteomic and R‐language analyses, highlighting the role of PSi NPs in the prognosis of the disease.

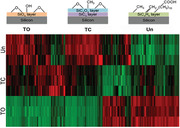

The prevailing investigation and application of nanoparticles (NPs) rapidly revolutionized many areas, especially, biomedicine technology. Whilst the perpetual effort in engineering different nanosystems with therapeutic advantages, another pivotal factor for their successful clinical translation is the thorough understanding and manipulation of their biocompatibility and biological responses.^[^ ^1^ ^]^ Previous studies scrutinized biocompatibility and immunogenicity of various types of NPs under healthy condition,^[^ ^2^ ^]^ and with the deeper understanding of NP−biosystem interactions, there has been an increasing interest to explore the potential impact of the NPs under diseased conditions, which is more clinically relevant.^[^ ^3^ ^]^ For example, previous studies have demonstrated that the bio‐fate of NPs was altered under a diseased condition,^[^ ^4^ ^]^ and results in impinging on the disease prognosis.^[^ ^5^, ^6^ ^]^ Therefore, it is meritorious to ponder and deliberate the biological responses of NPs within lesion sites for a better mechanistic basis understanding.

The clinical translation of porous silicon (PSi) particles has been expanding at a high pace during the past 20 years.^[^ ^7^ ^]^ Several PSi based clinical trials have been conducted to treat hepatocellular carcinoma and pancreatic cancer, where PSi are locally administrated to targeted sites.^[^ ^8^ ^]^ However, previous studies have shown that the crosstalk between NPs and surrounding diseased tissue may improve the disease prognosis by reversely reprogramming the detrimental microenvironment, such as modulating the inflammatory polarization.^[^ ^6^, ^9^ ^]^ Similarly, we have previously discovered that the administration of porous silicon nanoparticles (PSi NPs) within inflammatory conditions might affect the disease outcome to different extents,^[^ ^10^, ^11^ ^]^ despite their satisfactory biocompatibility and immunogenicity under healthy animal models.^[^ ^12^ ^]^ Yet, the rationale behind this phenomenon remains elusive. Therefore, a systematic study on investigating the potent biological response to PSi NPs under diseased condition may not only accelerate the clinical translation of PSi NPs, but also provide insights to further understand the bio–nano interactions.

A large interest and a plethora of previous studies have been reported to investigate the effect of the bio–nano interactions in lesion sites.^[^ ^13^, ^14^ ^]^ With the advent of proteomic investigation, the protein corona complex around the NPs has proven to be a critical factor in explaining this phenomenon.^[^ ^15^ ^]^ This extended paradigm proposes that protein corona formation under a diseased condition has a role not only in altering the biocompatibility and pharmacokinetics properties of NPs, but also interfering with the surrounding biological microenvironment.^[^ ^16^ ^]^ Based on this strategy, previous studies applied a variety of different NPs as a “nano‐concentrator” to exert both diagnostic^[^ ^13^, ^17^ ^]^ and therapeutic functions.^[^ ^18^, ^19^ ^]^ Yet, systematic investigations are still relatively less for aligning the particular parameters of nanomaterials in determining the effect on biological responses of NPs under diseased conditions, and to date there is no report in the literature demonstrating the corresponding information about PSi NPs.

In this study, we purpose that PSi NPs administration under conditions of acute liver inflammation (ALI, pre‐induced) could attenuate the liver necrosis and inflammatory response. We determined the importance of three critical parameters: surface hydrophobicity, porosity, and surface reducibility of PSi NPs. Herein, we propose that the inflammation attenuation capability of PSi NPs under ALI condition is positively correlated with the protein corona formation process, and this protein scavenging efficiency is more correlated with the porosity, rather than surface hydrophobicity of the NPs, whereas the reductive bonds within PSi NPs show minimal effects on attenuating the inflammation. The detailed composition of protein corona composition of each type of PSi NPs under ALI condition is confirmed by tandem‐mass‐tag (TMT) assisted proteomic study and the following R‐language facilitated analysis. In contrast to earlier works, which usually struggled to quantify hundreds to few thousands proteins, we extended this number to over 4000 types of proteins from each type of PSi corona complex, as such may provide deeper insight into the corona proteome and the bio–nano interactions under diseased condition. In conclusion, we identify the role of the protein corona in determining the immunogenicity of PSi NPs under an ALI condition, highlighting the role of PSi NPs in affecting the prognosis of the disease.

Three different PSi NPs with distinguished surface properties were produced, namely thermal oxidized PSi NPs (TO), thermal carbonized PSi NPs (TC), and undecylenic acid modified thermal hydrocarbonized PSi NPs (Un). They separately represent the majorly applied surface stabilizing methods, and obtain distinguished surface hydrophobicity properties (from hydrophilic to hydrophobic: TO, TC, Un). The difference of the surface hydrophobicity was confirmed through water contact angle (WCA) by placing a 5 µL droplet of water on a glass slide covered with dried PSi NPs film. Un obtained the most hydrophobic surface with WCA of 122° ± 6° compared to 62° ± 4° of TC and of 51° ± 5° of TO (**Figure** **1**a, Table S1, Supporting Information). Transmission electron microscopy (TEM) showed the morphology of different PSi NPs (Figure 1b). The hydrodynamic size and surface zeta‐potential of different PSi NPs were characterized with dynamic light scattering (DLS) coupled zeta‐potential analyzer, while their corresponding porosity was studied using N_2_ adsorption/desorption method. These NPs represent the commonly applied surface stabilization methods to obtain a similar size, zeta‐potential, and porosity (Table S1, Figure S1a, Supporting Information), but distinguished surface chemistries.^[^ ^20^, ^21^, ^22^ ^]^ Fourier transform infrared spectroscopy was conducted to confirm the surface chemistries of different PSi (Figure S1b, Supporting Information). Bands from TO were found at 3740 and 882 cm^−1^, which were respectively attributed to Si—OH and —O*_y_*Si—H*_x_*.^[^ ^20^ ^]^ The typical *ν*(C = O) band at 1715 cm^−1^ from Un confirmed the successful undecylenic acid hydrosilylation,^[^ ^22^ ^]^ and this band was also visible from TC, which was due to the acetylene treatment and the following high temperature from the annealing process.^[^ ^21^ ^]^ Hydrides (—O*_y_*Si—H*_x_*) remaining on the surface of the NPs can be confirmed by the PSi hydride stretch region at 2100–2300 cm^−1^.^[^ ^23^ ^]^ Further colloidal stability test was conducted in human plasma and the results confirmed that all three type of PSi NPs obtained a satisfied plasma stability within 1.5 h (Figure S1c,d, Supporting Information).

**Figure 1:**
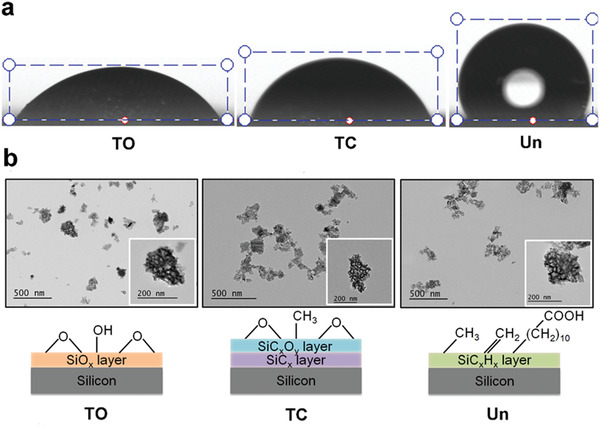
Characterization of different PSi NPs. a) Contact angle of water droplet on different PSi NPs film. Glass slides were covered with different types of dried PSi NPs to form into PSi NPs film. The WCA of different surface chemistries was estimated by placing a 5 µL droplet of water on corresponding PSi film, photograph of the droplet was taken and analyzed with Attension Theta Optical Tensiometer. The water contact angle of different PSi confirmed the surface hydrophobicity properties of each type of NPs where TO is the most hydrophilic NPs and Un is the most hydrophobic NPs. b) Graphic illustration of the surface chemistries of the different PSi NPs and the corresponding TEM images. The TEM images confirmed the porous structure of each type of PSi NPs and the observed morphology and size are in consistence with their corresponding hydrodynamic size obtained via DLS method.

We found that PSi NPs mainly accumulate in liver when it is administrated intravenously.^[^ ^10^ ^]^ Therefore, to better investigate the immunomodulatory effects of different PSi NPs within pre‐existing lesion sites, ALI was induced in mice by administration of acetaminophen (APAP). High dose of APAP results in hepatocellular necrosis within 1.5 h,^[^ ^24^ ^]^ which further amplifies the inflammatory process and then aggravates liver injury.^[^ ^25^ ^]^ 3 h post APAP administration, TO, TC, and Un at different concentrations (0.3 or 3 mg kg^−1^ as low (L) or high (H) concentration, respectively) were injected in ALI mice via i.v. Healthy or ALI mice without PSi NPs treatment were referred as healthy or ALI group. 48 h after PSi NPs administration, blood and tissues were collected for further study.

We first evaluated the systemic changes of each group. ALI group showed a significant increase in liver index, while the injection of PSi NPs had limited effects on both body weights and liver index compared to ALI group (Figure S2a,b, Supporting Information). In the hematologic test, ALI group showed a significantly enhanced total white blood cells (WBC) number (*p* = 0.013) in comparison with the healthy group, as a result of successful ALI establishment.^[^ ^26^ ^]^ Comparing to ALI group, none of the PSi NPs promoted any further increase in the WBC number (**Figure** **2**a). Similarly, no significant WBC composition changes were observed after PSi administration since the percentage of three main types of WBC, namely granulocytes (Figure 2b), monocytes (Figure 2c), and lymphocytes (Figure 2d), remained comparable to ALI group. In addition, hematoxylin and eosin (H&E) staining and TdT‐mediated dUTP Nick‐End labeling of liver sections also suggest that administration of PSi in ALI mice did not exacerbate liver damage (Figure S3a,b, Supporting Information). Interestingly, the serum biochemical analysis showed a significant decrease (Figure 2f) of the aspartate aminotransferase (AST) value for most of the PSi‐treated mice compared to the ALI group, while alanine aminotransferase (ALT) value was modestly affected (Figure 2e), resulting into an overall reduced AST/ALT value, which was statistically different in the Un H group (Figure 2g). A similar effect was observed for the alkaline phosphatase (ALP), where administration of high concentration Un showed a significant decrease comparing to ALI group (Figure 2h). Moreover, the injection of TO and Un at high concentration also led to an enhanced glutathione (GSH) level in mice suffering from ALI (*p* = 0.032 for TO H and *p* = 0.019 for Un h, Figure 2i). Overall, PSi NPs injection under an ALI condition slightly reduces liver injurious extents, where TO and Un had an overall better capability comparing to TC.

**Figure 2:**
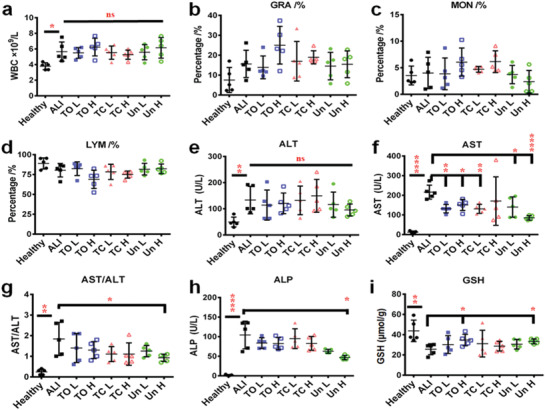
Administration of PSi NPs affected the disease outcome of ALI. a) Administration of PSi NPs under an inflammatory condition, showing limited effect on secondary WBC number enhancement. In vivo blood immune cell alterations after ALI establishment and PSi NPs administration. Blood samples were collected, as described above. Percentage of three main types of leukocytes, namely b) monocytes, c) granulocytes, and d) lymphocytes, in total WBC were analyzed. e) Serum ALT as a measure of liver injury. PSi administration under ALI condition showed no significant effect on neither deteriorating nor attenuating ALT value. f) Determination of serum AST level from different groups, where TO L, TO h, TC L, Un L, and Un H were statistically significant different. g) Yielded AST/ALT value of different group. Un H group reached statistical significance. h) Serum ALP value of Un H group was significantly lower than ALI group. i) The hepatocellular GSH value from TO H and Un H group were significantly higher than ALI group. All data in the graphs are shown as mean ± SD from five independent replicates. *p < 0.05, **p < 0.01, ***p < 0.0005, and ****p < 0.0001 compared with ALI group (two‐tailed Student's *t*‐test).

We hypothesized the improved disease outcome of PSi NPs can be mainly attributed to the subsequent pro‐inflammatory cascade responded to hepatocellular necrosis.^[^ ^24^, ^25^, ^27^ ^]^ To confirm this, quantitative real‐time polymerase chain reaction (qPCR) was applied to quantify the mRNA expression level associated with key pro‐inflammatory cytokines and chemokines, including IL‐1*β*, IL‐6, tumor necrosis factor *α* (TNF‐*α*), chemokine (C‐X‐C motif) ligand 1 (CXCL‐1), and chemokine (C‐C motif) ligand 2 (CCL‐2). Comparing to a healthy condition, mice suffering from ALI exhibited 2–5‐folds higher mRNA expression for the indicated cytokines and chemokines. However, PSi administration showed a surface chemistry dependent immunomodulatory effect in the inflammatory condition, where TO (H) group significantly reduced the TNF‐*α* and IL‐6 expression, whilst Un (H) group reduced TNF‐*α*, IL‐6, and IL‐1*β* expression, yet TC at both concentrations showed a sparing effect (**Figure** **3**a). This was in consistence with the aforementioned results that TO (with the most hydrophilic surface) and Un (with the most hydrophobic surface) showed a more significant role in reducing the inflammatory response comparing to TC.

**Figure 3:**
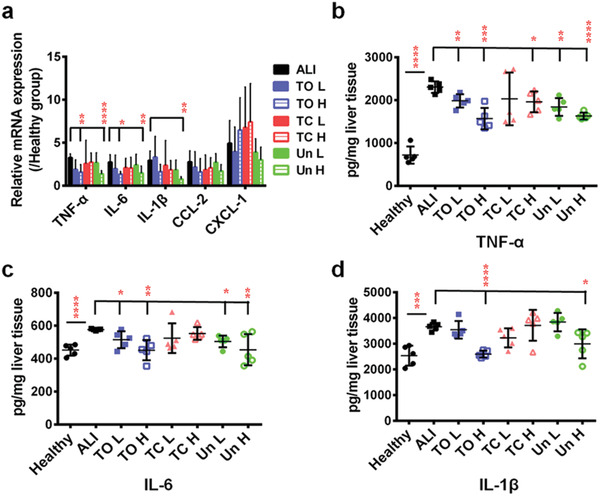
Immuno‐modulation effects of different PSi NPs under ALI condition. a) Effect of PSi NPs administration on pro‐inflammatory cytokines mRNA synthesis. The mRNA expression levels of each cytokines from healthy mice were fixed as 1. TO at high concentration could reduce the TNF‐*α* and IL‐6 mRNA expression, whereas Un at high concentration could reduce TNF‐*α*, IL‐6, and IL‐1*β* mRNA expression comparing to ALI group. Inhibition effect of PSi NPs on ALI‐mediated pro‐inflammatory cytokines (TNF‐*α*, IL‐6, and IL‐1*β*) release. b) TNF‐*α* production could be reduced by TO L, TO h, TC h, Un L, and Un H treatment; c) IL‐6 production could be reduced by TO L, TO h, Un L, and Un H treatment; d) IL‐1*β* production could be reduced by TO h, TC L, and Un H treatment. All data in the graphs are shown as mean ± SD from five independent replicates. *p < 0.05, **p < 0.01, ***p < 0.0005, and ****p < 0.0001 compared with ALI group (two‐tailed Student's *t*‐test).

Enzyme linked immunosorbent assay (ELISA) was conducted to quantify the amount of three main pro‐inflammatory cytokines (TNF‐*α*, IL‐6, and IL‐1*β*) in the liver. Similarly, TO and Un administration downregulated all three cytokines production in a PSi NPs’ concentration dependent manner, whereas this phenomenon from TC was marginally present, as TC at low concentration slightly reduced the IL‐1*β* production, while high concentration of TC only slightly inhibited TNF‐*α* production (Figure 3b–d). Taken altogether, the phenomenon of PSi surface dependent inflammation attenuation can partly explain the above‐mentioned injury mitigation. However, this inflammation mitigation cannot be linearly correlated with the hydrophobicity of the PSi surfaces.

Since the appearance or increase of acute phase proteins in plasma, which comprises mostly pro‐inflammatory signaling proteins, is one of the prominent features for ALI,^[^ ^28^ ^]^ we wonder whether the interaction between plasma proteins and different PSi NPs would account for the surface dependent inflammatory attenuation. We first evaluated the different protein binding pattern from different PSi NPs. Blood plasma from ALI and healthy mice was collected and incubated with different PSi NPs to form the protein corona. Then, the whole plasma proteins from healthy mice, ALI mice and proteins in corona were separated by sodium dodecyl sulphate‐polyacrylamide gel electrophoresis. A macroscopic difference in protein‐binding pattern was observed from different PSi protein corona (Figure S4a,b, Supporting Information), and relative intensity of each bands zone (relative densitometry) was calculated to evaluate this difference in a more detailed manner (Figure S4c, Supporting Information), which confirmed an altered protein corona composition from different PSi NPs.

To further confirm that PSi with different surface chemistries altered protein binding capability, we investigated whether PSi might potentially associate with pro‐inflammatory proteins, such as cytokines, and we separately evaluated the absorption of three main pro‐inflammatory cytokines (TNF‐*α*, IL‐1*β*, and IL‐6) to different PSi NPs. Different PSi NPs were separately incubated with TNF‐*α*, IL‐1*β*, and IL‐6 stock solution and the detailed cytokine binding capability was measured through ELISA method. Results suggested different from TC and Un, only TO obtained a concentration dependent absorption capability for IL‐1*β*, whereas Un exhibited a statistically significant IL‐6 absorption (Figure S4d, Supporting Information). These results indicated that different PSi NPs had a prominent discrepancy between protein binding patterns. However, the results have not been conclusive enough for an understanding of the correlation between this difference and the biological phenomenon.

As such, we further adapted a quantitative proteomic method to unravel the detailed composition of the protein corona from each type of PSi NPs. Isobaric tags for TMT method is particularly useful for comparative quantitative analysis, and enables more accurate and multiplexed quantification comparing to conventional methods, such as isotope coded affinity tags procedure.^[^ ^29^, ^30^ ^]^ Different PSi NPs were incubated with plasma extracted from ALI mice, and the proteins from the corona‐complex were further eluted for analysis, as described previously with slight modification.^[^ ^30^ ^]^ As a result, we obtained a large proteome datasets of 4459 types of proteins identified from all three types of corona complex, which is significantly higher than previous reports.^[^ ^31^ ^]^ The amount of the database is the critical prerequisite for successful and accurate data mining, and therefore, this enhanced number in identified proteins will further facilitate the following R‐language based data analysis. Among the totally identified types of proteins, 2123 types of proteins with *p* < 0.05 were considered to obtain a significantly altered affinity toward different PSi based on the one‐way analysis of variance (ANOVA) comparison. Further analysis was followed by a Student's *t*‐test, and proteins with the *p* < 0.05 were selected and classified by their major enrichment (given as “TO,” “TC,” or “Un”). Among which, 1127 of them were mainly captured by TO, whereas the corresponding number for TC and Un were 483 and 513, separately (Data S1, Supporting Information). The proteins enriched in each group were then subjected to Gene Ontology analysis using the database for annotation, visualization, and integrated Discovery. R‐language analysis was further applied to explore the protein binding pattern of different PSi NPs, these proteins were first classified by their subcellular location (**Figure** **4**a). Results suggested that all types of PSi captured massive intracellular proteins that were usually not observed in healthy plasma. Noteworthy, the percentage of mitochondrial proteins within the TO‐corona complex (22.8%) was significantly higher than TC‐corona (13.9%) and Un‐corona (12.5%), whereas the most distinguished feature for Un‐corona was the high percentage of extracellular proteins within its corona complex (TO‐corona, 15.1%; TC‐corona, 11.0%; Un‐corona, 28.5%).

**Figure 4:**
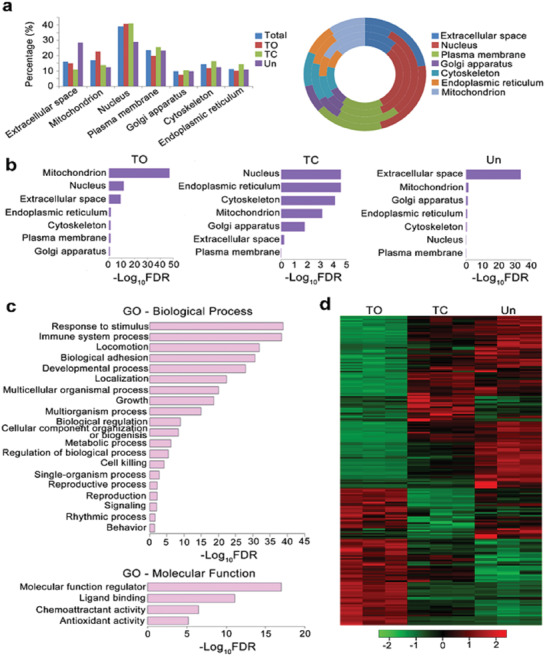
Quantification and comparison of different corona‐complex identified by TMT and the following R‐language facilitated analysis. a) Proteins significantly absorbed by TO, TC, and Un identified and further classified by their subcellular location. Percentage of proteins from different subcellular locations shown as the column chart (left panel) and doughnut chart (right panel). For the doughnut chart, the corresponding annulus represented total, TO, TC, and Un (from inside to outside), respectively. b) Biased protein absorption pattern from different PSi NPs, GO annotation investigated based on the CC term. c) The GO term enrichment of proteins identified from NPs’ corona complex and classified as extracellular/secreted according to their subcellular location. Biological processes of GO terms and molecular functions of GO terms were separately investigated. d) In total 244 types of extracellular/secreted proteins annotated as inflammation‐related proteins and a heat map further plotted according to the relative amount of each protein in different NPs’ corona complex, indicating a distinguished extracellular/secreted proteins binding pattern to different PSi NPs. Log‐transformed normalized peak intensities of proteins in each samples were expressed using red, black, or green colors in a heat map (*n* = 3).

Gene ontology (GO) analysis was further conducted based on cellular component (CC) to demonstrate the biased proteins binding pattern of different PSi. GO terms with false discover rate (FDR) < 0.05 were considered as significant enrichment. As shown in Figure 4b, for the proteins mainly captured by TO, the most significant enrichment of CC is in mitochondrion (GO: 0005739, FDR = 7.49 × 10^−48^), suggesting TO had propensity toward binding mitochondrial proteins. Despite TC and Un did not obtain distinct affinity toward any specific type of intracellular proteins, Un showed a distinguished enrichment in absorbing extracellular proteins (GO: 0005615, FDR = 1.81 × 10^−34^).

In total, 862 extracellular and secreted proteins were further filtered (Data S2, Supporting Information), and then subjected to biological process (BP) and molecular function (MF) annotations to obtain their biological roles. Among which, response to stimulus (GO: 0050896, FDR = 1.50 × 10^−39^), immune system process (GO: 0002376, FDR = 4.40 × 10^−39^), and locomotion (GO: 0040011, FDR = 1.30 × 10^−32^) were the most significant enrichments of biological process; molecular function modulator (GO: 0098772, FDR = 9.60 × 10^−18^), ligand binding (GO: 0005488, FDR = 1.30 × 10^−14^), and chemoattractant (GO: 0042056, FDR = 3.50 × 10^−7^) were the most significant enrichments of molecular function (Figure 4c). These results suggested the pro‐inflammatory properties of the captured proteins within the corona‐complex.

Within the overall 862 extracellular/secreted proteins, 244 types of proteins were directly associated with inflammatory cascade and obtained enough abundance in all three types of corona‐complex for conducting relative quantification. This subset of proteins not only contained the pro‐inflammatory cytokines and/or chemokines, but also massive damage‐associated molecular patterns (DAMPs), such as high mobility group protein B1 or heat shock proteins, which are commonly enriched in damaged or inflamed tissues and further amplify the inflammatory process.^[^ ^32^ ^]^ To discover PSi surface chemistry specific protein absorption pattern, one‐way ANOVA was performed. Within the 244 types of extracellular immune‐regulatory proteins, 208 identified proteins with *p* < 0.05 were considered to obtain a significantly altered affinity between different PSi, as shown in the heat map. By comparing relative abundance, 84 types of proteins were mainly enriched in TO‐corona and the corresponding number for TC‐corona and Un‐corona were 33 and 91 (Figure 4d, Data S2, Supporting Information). Overall, these results indicate a surface‐based plasma protein absorption pattern of different PSi NPs. Among which, TO obtained the highest affinity toward mitochondrial proteins, which is the major source of DAMPs‐induced inflammation, whereas Un showed better capturing ability toward pro‐inflammatory signaling proteins, and this specific binding pattern might account for the improvement of ALI prognosis.

To further confirm our hypothesis, we next checked whether this protein scavenge induced immune‐modulation was feasible in vitro. Murine macrophage cell line RAW 264.7 were incubated with Dulbecco's modified Eagle's medium (DMEM) containing 10% of healthy mice plasma (further referred as healthy medium) or ALI mice plasma (referred as ALI medium). As a key signaling pathway for modulating inflammation, immunoblot analysis of nuclear factor *κ*B (NF‐*κ*B) pathway was carried out to evaluate the immunomodulatory effect of PSi in presence of ALI plasma. As shown in **Figure** **5**a, the pro‐inflammatory effect of ALI plasma was confirmed as ALI medium enhanced the phosphorylation of NF‐*κ*B inhibitor, inhibitor *κ*‐B*α*, upregulating the expression of NF‐*κ*B subunit transcription factor p65 (RELA), rendering a pro‐inflammatory cascade. However, ALI medium pre‐incubated with TO or Un, but not TC, showed a decreased NF‐*κ*B activation comparing to blank ALI medium (Figure S5a, Supporting Information)

**Figure 5:**
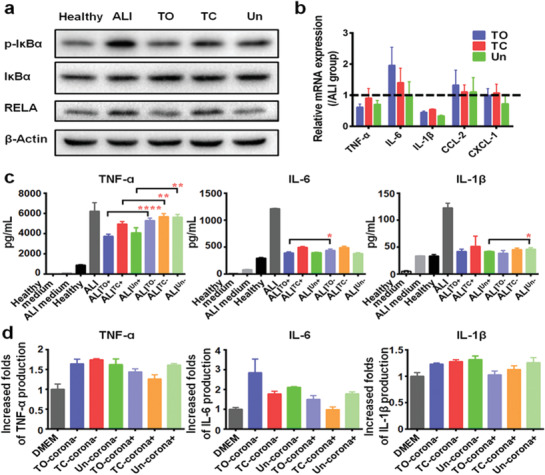
PSi treatment can modulate the immunostimulative effect of ALI plasma through protein corona formation. a) TO and Un downregulate ALI plasma induced NF‐*κ*B activation in macrophages. RAW 264.7 macrophages cells treated 1 h with healthy medium, ALI medium, ALI medium pre‐treated and containing 20 µg mL^−1^ of different PSi NPs were subjected to immunoblotting assay. b) Treatment with PSi NPs attenuated ALI plasma induced pro‐inflammatory cytokines/chemokines mRNA synthesis. RAW 264.7 cells treated 1.5 h with ALI medium, ALI medium pre‐treated and containing 20 µg mL^−1^ of different PSi NPs. The mRNA expression levels of each cytokines from RAW 264.7 macrophages cells treated with blank ALI medium were fixed as 1. c) Pro‐inflammatory cytokines’ secretion profile of macrophages treated with healthy medium, ALI medium, ALI medium containing 20 µg mL^−1^ of different PSi NPs (ALI^TO+^, ALI^TC+^, and ALI^Un+^) and ALI medium pre‐incubated, but without containing 20 µg mL^−1^ of different PSi NPs (ALI^TO−^, ALI^TC−^, and ALI^Un−^) for 24 h. Values are expressed as the means ± SDs of four independent trails. Statistical significance is assessed by two‐tailed Student's *t*‐test; *p < 0.05, **p < 0.01, ***p < 0.0005, and ****p < 0.0001. d) 20 µg mL^−1^ of different PSi NPs incubated with ALI medium for 4 h, followed by centrifugation to recover the pellet. Macrophages were treated with blank DMEM, DMEM containing 20 µg mL^−1^ pristine PSi (TO‐corona^−^, TC‐corona^−^, and Un‐corona^−^) or DMEM containing 20 µg mL^−1^ recovered PSi pellets (TO‐corona^+^, TC‐corona^+^, and Un‐corona^+^) after 24 h, and then the cytokines’ concentration in the supernatant measured afterward. The cytokine excretion level from DMEM group were fixed as 1. Values are expressed as the means ± SDs of four independent trails.

The mRNA expression level associated with key pro‐inflammatory cytokines/chemokines were also evaluated via qPCR. As shown in Figure S5b, Supporting Information, cells treated with ALI medium exhibited a time‐dependent elevation in cytokines/chemokines expression and reached to peaks at 1.5 h, except CXCL‐1 with a peak at 2.5 h. We next examined whether NPs’ corona formation might impact on the pro‐inflammatory cytokines/chemokines’ mRNA expression. ALI medium was pre‐incubated with different PSi at the concentration of 20 µg mL^−1^ for 4 h, then the PSi NPs containing ALI medium were incubated with RAW 264.7 macrophage cells for another 1.5 h, while blank ALI medium was used as control. As shown in Figure 5b, PSi treatment modulated the pro‐inflammatory effect of ALI medium in different extent, where TO showed an enhanced IL‐6 expression, but a decreased IL‐1*β* expression; TC only reduced IL‐1*β* expression and Un downregulated the expression of TNF‐ɑ and IL‐1*β*. In particular, this phenomenon was likely induced by the ALI plasma absorption as blank PSi NPs administration induced a different expression profile of these genes (Figure S5c, Supporting Information).

Further ELISA assays were carried out to decipher the detailed immune‐modulation functions of captured proteins in corona‐complex by quantitatively elucidating the cytokines release profile. RAW 264.7 macrophage cells were separately treated with healthy medium (Healthy); ALI medium (ALI); ALI medium containing 20 µg mL^−1^ of TO (ALI^TO+^), TC (ALI^TC+^), and Un (ALI^Un+^); ALI medium pre‐treated with 20 µg mL^−1^ of TO, TC, and Un for 4 h, followed by removing the NPs via centrifugation (ALI^TO−^, ALI^TC−^, and ALI^Un−^) after 24 h. Comparing to Healthy group, ALI group showed 7.0‐fold of TNF‐*α*, 4.1‐fold of IL‐6, and 3.7‐fold of IL‐1*β* excretion, confirming the pro‐inflammatory effects of plasma from ALI mice. PSi treatment of ALI medium pervasively ameliorated its pro‐inflammatory effect, and the general inflammatory attenuation effect from different PSi was TO ≈ Un > TC. As comparing to ALI group, ALI^TO+^ reduced 40% of TNF‐*α* production, 68% of IL‐6 production, and 66% of IL‐1*β* production. The corresponding number for ALI^TC+^ was 20%, 59%, and 58%, and the number for ALI^Un+^ was 34%, 66%, and 66%. Moreover, ALI^TO−^, ALI^TC−^, and ALI^Un−^ also subsided inflammation activation. Besides of TNF‐*α*, they exhibited an overall similar cytokines’ production profile comparing to ALI^TO+^, ALI^TC+^, and ALI^Un+^, suggested inhibited pro‐inflammatory activities was likely due to pro‐inflammatory proteins adsorption onto the particle surfaces, as proposed in the previous section (Figure 5c).

Previous studies reported the formation of protein corona on NPs could either stimulate or suppress inflammatory responses in immune cells.^[^ ^33^, ^34^ ^]^ To test the role of the PSi‐corona complex in determining the immunogenicity of PSi under ALI condition, RAW 264.7 cells were separately treated with FBS free DMEM medium (DMEM); FBS free DMEM medium containing 20 µg mL^−1^ TO (TO‐corona+), TC (TC‐corona+), and Un (Un‐corona+) recovered from ALI medium treatment; FBS free DMEM medium containing pristine TO (TO‐corona‐), TC (TC‐corona‐), and Un (Un‐corona‐) for 24 h. Results suggest PSi‐corona complex exhibited non‐superior cytokines production comparing to pristine PSi, and TO‐corona^+^ and TC‐corona^+^ even showed less pro‐inflammatory stimulation effects comparing to their corresponding counterparts. Moreover, TO‐corona^+^, TC‐corona^+^, and Un‐corona^+^ only obtained limited immunostimulatory effects, as comparing to DMEM group, TO‐corona^+^ exhibited 1.4‐, 1.5‐, and 1.0‐fold higher production for TNF‐*α*, IL‐6, and IL‐1*β*, and the corresponding number for TC‐corona^+^ were 1.3‐, 1.0‐, and 1.1‐fold, and for Un‐corona^+^ the respective number were 1.6‐, 1.8‐, and 1.3‐fold (Figure 5d). This suggest the limited immunogenicity of the bare PSi‐corona complex, further confirmed PSi‐absorption could block the biological activity of the pro‐inflammatory proteins. Overall, all these data suggest that the blood plasma collected from ALI mice initiated a pro‐inflammatory process, whereas this effect was modulated by PSi NPs due to the protein absorption and protein corona formation. And this protein binding pattern was dependent on the surface chemistry properties of PSi NPs.

The above‐mentioned results suggested that PSi NPs can affect the ALI prognosis through absorbing inflammatory‐stimuli proteins in plasma under a diseased condition. However, this inflammation attenuation efficiency is not dictated by the surface hydrophobicity of the PSi NPs, as different PSi NPs obtained a significantly altered protein binding pattern, and no linear correlation between the hydrophobicity of the NPs and the inflammatory‐stimuli protein binding was revealed through the R‐language based machine analysis. To further explore the pivotal factor of NPs in determining this phenomenon, we hypothesized whether PSi NPs, with the same surface chemistry but different protein binding capability, could show different inflammation modulation efficiency. To confirm this, we separately synthesized a new batch of TO, TC, and Un NPs (N‐TO, N‐TC, and N‐Un) with significantly reduced porosity (including specific surface area, total pore volume and average pore diameter) compared to the conventional PSi NPs (Table S2, Supporting Information). The BCA assay confirmed that TO, TC and Un NPs obtained higher protein binding capability compared to their corresponding counterparts (N‐TO, N‐TC, and N‐Un, Figure S6, Supporting Information). ELISA assays were further carried out to compare the immune‐modulation functions of two different batches of PSi NPs. RAW 264.7 macrophage cells were separately treated with ALI medium (ALI); ALI medium containing 20 µg mL^−1^ of TO (ALI^TO+^), N‐TO (ALI^NTO+^), TC (ALI^TC+^), N‐TC (ALI^NTC+^), Un (ALI^Un+^), and N‐Un (ALI^NUn+^); ALI medium pre‐treated with 20 µg mL^−1^ of TO, N‐TO, TC, NTC, Un, and N‐Un for 4 h, followed by removing the NPs via centrifugation (ALI^TO−^, ALI^NTO−^, ALI^TC−^, ALI^NTC−^, ALI^Un‐^, and ALI^NUn−^) after 24 h. Consistent with the aforementioned results, all types of PSi NPs treatment attenuated the ALI plasma induced inflammation stimulation in different extents. Yet, the results showed that the new batch of PSi NPs showed an overall lower inflammation attenuation efficiency compared to their corresponding counterparts with higher porosity and protein binding capability (**Figure** **6**). This further consolidate our previous hypothesis that the “protein sweep” capability of PSi NPs may be responsible for the inflammation attenuation phenomenon under an ALI condition. Whereas this protein binding affinity is positively correlated with the porosity of PSi NPs, thus may further dictate the inflammation attenuation efficiency in ALI.

**Figure 6:**
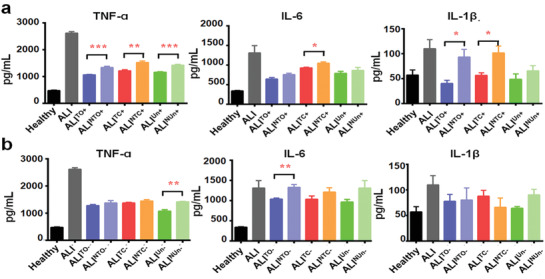
PSi with less porosity showed less immunostimulative modulation effect on ALI plasma. a) Pro‐inflammatory cytokines’ secretion profile of macrophages treated with healthy medium, ALI medium, ALI medium containing 20 µg mL^−1^ of TO, TC, Un (ALI^TO+^, ALI^TC+^, ALI^Un+^) or their corresponding counterparts with less porosity but same surface chemistry (ALI^NTO+^, ALI^NTC+^, ALI^NUn+^). b) Pro‐inflammatory cytokines’ secretion profile of macrophages treated with healthy medium, ALI medium, ALI medium pre‐incubated, but without containing 20 µg mL^−1^ of different PSi NPs (ALI^TO−^, ALI^NTO−^, ALI^TC−^, ALI^NTC−^, ALI^Un‐^, and ALI^NUn−^) for 24 h. Values are expressed as mean ± SEM of four independent trails. Statistical significance is assessed by two‐tailed Student's *t*‐test; *p < 0.05, **p < 0.01, and ***p < 0.0005.

We were asked whether the reductive nature of PSi NPs may be responsible for ameliorating inflammation, as despite the surface stabilization, the major part of PSi NPs is still composed by reductive Si—Si bonds. And the reductive silicon bonds may consume and scavenge ROS under inflammatory condition to proceed to oxidation process, further facilitate the hydrolysis and degradation (Figure S7a, Supporting Information),^[^ ^4^, ^35^ ^]^ as previous reports demonstrated the physiochemical nature of NPs may also partly determine their immunogenicity.^[^ ^33^, ^36^ ^]^ Indeed, the co‐incubation of PSi NPs and H_2_O_2_ can reduce the H_2_O_2_ amount in a PSi concentration dependent manner (Figure S9a, Supporting Information). TO showed the most prominent scavenging efficiency, whereas Un had a negligible effect, and the ranking for scavenge effects from different PSi was TO > TC > Un. This might be caused by the hydrophobic surface of TC and Un preventing the surface wetting process, which was confirmed by the WCA results (Table S1, Supporting Information). We further observed the degradation kinetics of different PSi with or without addition of extra ROS. As shown in Figure S7b, Supporting Information, addition of ROS accelerated the PSi degradation in different extent, and adding ROS into TO could enhance degradation within 6 h, yet this phenomenon from TC was not statistically different until after 2 days, and Un only showed a statistical significant difference at day 7. This could also be explained as, the different hydrophobicity from TO, TC and Un determined the NPs surface wetting efficiency, this may not only dictate the interaction between ROS and the Si back bonds, but also affect the following hydrolysis process.

ROS scavenge phenomenon was further investigated in RAW 264.7 cells and the ROS levels were monitored by 2',7'‐dichlorodihydrofluorescein diacetate fluorescence assay^[^ ^37^ ^]^. In consistent with previous reports, TO, TC, and Un showed low to no effects on the cellular viability of RAW 264.7 cells (Figure S8a, Supporting Information).^[^ ^12^ ^]^ Under normal condition, addition of TO, TC, and Un, independent of the NPs’ concentration, showed no statistical significant effect on the intracellular ROS levels (Figure S7c, Supporting Information), which is consistent with the previous report^[^ ^37^ ^]^. We then evaluated the intracellular ROS perturbation effect of PSi NPs under an inflammatory condition with lipopolysaccharide and interferon *γ* cotreatment, which simulated the pro‐inflammatory condition, triggered more than twofold of ROS generation in RAW 264.7 cells. However, the sequential administration of PSi showed low to no effect on the intracellular ROS content, suggesting that as observed ROS consumption effect from PSi was not feasible in vitro (Figure S7d, Supporting Information), and PSi NPs administration showed no effect on the cellular morphology of RAW 264.7 cells (Figure S8b, Supporting Information). This phenomenon was further confirmed by the viability test, where HepG2 liver carcinoma cells were treated by various concentrations of H_2_O_2_ for 24 h. Simultaneous coadding all types of PSi NPs with H_2_O_2_ did not reverse the H_2_O_2_ induced cellular apoptosis (Figure S9b,c, Supporting Information). However, when we pre‐incubated PSi with H_2_O_2_ containing medium for 24 h, followed by incubation of HepG2 cells with the corresponding medium, attenuated toxicity from the H_2_O_2_ was observed in treatment group of the medium pre‐treated with TO (Figure S7e, Supporting Information). Together, our results suggested that the ROS scavenge effect from PSi NPs was a rather slow process and has limited effect on modulating the intracellular ROS content in vitro.

Overall, using ALI mice model, we scrutinized the composition of protein corona around PSi NPs, followed by R‐language based molecular mechanism investigation, revealing the important role of such protein corona in modulating immunogenicity. These results were different from the observation under normal physiological conditions that TO and TC showed good biocompatibility, while Un exhibited mild liver toxicity.^[^ ^37^, ^38^ ^]^

Under ALI condition, acute phase proteins, mostly pro‐inflammatory signaling proteins, are abnormally secreted in plasma.^[^ ^27^ ^]^ Furthermore, massive intracellular proteins, which are leaked to plasma after cellular damage and further function as natural endogenous adjuvants to induce phlogosis,^[^ ^39^ ^]^ and Un and TO, comparing to TC, could better capture such kind of extracellular signaling proteins.

In summary, we have identified a significant role of protein corona under diseased conditions in regulating the immunogenicity of PSi NPs. More importantly, this immunological impact of PSi NPs is minimal related with the reductive nature of PSi NPs, but more governed by their porosity, rather than surface hydrophobicity. However, we should be noted that despite the fact that we failed to find any liner correlation between surface hydrophobicity of NPs and the pro‐inflammatory protein binding capability, it has long been unraveled the pivotal role of surface chemistry of NPs on dictating the specific protein binding.^[^ ^19^, ^40^ ^]^ Therefore, further efforts should be made to achieve a “designed adsorption” via NPs with specific surface chemistries.^[^ ^41^ ^]^ This may provide insights for designing PSi NPs based pharmaceutical products, further facilitate its clinical translation, meanwhile offering alternative perspective for evaluating the biocompatibility of other NPs in pathological circumstances.

## Experimental Section

All the experimental details are reported in the Supporting Information. A list of abbreviations and variations are listed in Table S3. All experiments involving animals were performed in compliance with the guidelines from the Institutional Animal Care and Use Committee at Experimental Animal Centre in Xiamen University, China.

## Conflict of Interest

The authors declare no conflict of interest.

## Supporting information

Supporting Information

Supporting data1

Supporting data2
