# Correction to “Tandem‐Mass‐Tag Based Proteomic Analysis Facilitates Analyzing Critical Factors of Porous Silicon Nanoparticles in Determining Their Biological Responses under Diseased Condition”

**DOI:** 10.1002/advs.202509004

**Published:** 2025-07-17

Adv. Sci. 2020, 7, 2001129.

https://doi.org/10.1002/advs.202001129

We noticed an unintentional error in Figure S8b, specifically in the images for the “TO” and “TC” groups at the 48 h time point. It appears that the same image was mistakenly duplicated for these two conditions.

The wrong images were inserted by mistake with no intent to obscure or alter any results. Furthermore, the corresponding quantitative data (Figure S8a, Supporting Information) confirm that none of the tested conditions (Un, TC, and TO) significantly affect RAW 264.7 macrophage viability. Therefore, although the images need to be corrected, the overall findings and conclusions remain valid.

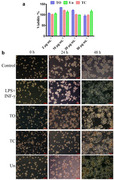

We apologize for this error.

## Supporting information



Supporting Information

