# Supplementary material for: Tandem‐Mass‐Tag Based Proteomic Analysis Facilitates Analyzing Critical Factors of Porous Silicon Nanoparticles in Determining Their Biological Responses under Diseased Condition
Source: Adv Sci (Weinh). 2020 Jun 23;7(15):2001129. doi: 10.1002/advs.202001129 (PMC7404168; doi:10.1002/advs.202001129)
Supplement: Supplementary file 1 — Supporting Information [file ADVS-7-2001129-s001.pdf]

© 2020 Wiley-VCH GmbH

ADVANCED SCIENCE Open Access

Supporting Information

for *Adv. Sci.*, DOI: 10.1002/advs.202001129

Tandem-Mass-Tag based proteomic analysis facilitates analyzing critical factors of porous silicon nanoparticles in determining their biological responses under diseased condition

Yunzhan Li, Zehua Liu, Li Li, Wenhua Lian, Yaohui He, Elbadry Khalil, Ermei Mäkilä, Wenzhong Zhang, Giulia Torrieri, Xueyan Liu, Jingyi Su, Yuanming Xiu, Flavia Fontana, Jarno Salonen, Jouni Hirvonen, Wen Liu, Hongbo Zhang, Hélder A. Santos*, and Xianming Deng**

Copyright WILEY-VCH Verlag GmbH & Co. KGaA, 69469 Weinheim, Germany, 2020.

Supporting Information

Tandem-Mass-Tag based proteomic analysis facilitates analyzing critical factors of porous silicon nanoparticles in determining their biological responses under diseased condition

*Yunzhan Li, Zehua Liu, Li Li, Wenhua Lian, Yaohui He, Elbadry Khalil, Ermei Mäkilä, Wenzhong Zhang, Giulia Torrieri, Xueyan Liu, Jingyi Su, Yuanming Xiu, Flavia Fontana, Jarno Salonen, Jouni Hirvonen, Wen Liu, Hongbo Zhang**, Hélder A. Santos**, and Xianming Deng**

[*] Dr. Y.Z. Li, Dr. L. Li, W.H. Lian, X.Y. Liu, J.Y. Su, Y.M. Xiu and Prof. X. Deng
State Key Laboratory of Cellular Stress Biology, Innovation Center for Cell Signaling Network, School of Life Sciences, Xiamen University
361101, Fujian, China
E-mail: xmdeng@xmu.edu.cn

[*] Dr. Y.Z. Li, Dr. L. Li, W.H. Lian, X.Y. Liu, J.Y. Su, Y.M. Xiu and Prof. X. Deng
State-Province Joint Engineering Laboratory of Targeted Drugs from Natural Products, School of Life Sciences, Xiamen University
361101, Fujian, China

[*] Dr. Z.H. Liu, E. Khalil, G. Torrieri, Dr. F. Fontana, Prof. J. T. Hirvonen and Prof. H. A. Santos
Drug Research Program
Division of Pharmaceutical Chemistry and Technology, Drug Research Program
Faculty of Pharmacy, University of Helsinki
FI-00014, Helsinki, Finland
E-mail: helder.santos@helsinki.fi

[*] Prof. H. A. Santos
Helsinki Institute of Life Science (HiLIFE)

University of Helsinki
FI-00014, Helsinki, Finland

[*] Prof. H. Zhang
Department of Pharmaceutical Science, Åbo Akademi University
FI-20520, Turku, Finland
E-mail: hongbo.zhang@abo.fi

[*] Prof. H. Zhang
Turku Center of Biotechnology, Åbo Akademi University
FI-20520, Turku, Finland

Y.H. He, Prof. W. Liu
School of Pharmaceutical Sciences, Xiamen University.
361101, Fujian, China

E. Mäkilä, Prof. J. Salonen
Laboratory of Industrial Physics, Department of Physics, University of Turku
FI-20014, Turku, Finland

Dr. W.Z. Zhang
Department of Chemistry, University of Helsinki
FI-00014, Helsinki, Finland

Keywords: R-language, protein corona, immunogenicity, porous silicon, acute liver inflammation

Contents

1. Experimental Section.

2. Table S1 Quantitative characterization of different PSi NPs.

3. Table S2 Quantitative porosity characterization of new batch of PSi NPs.

4. Table S3 List of abbreviation and variations.

5. Figure S1. Surface characterization of different PSi NPs.

6. Figure S2. Body weight and liver index of mice from each group.

7. Figure S3. Histological and immunohistological analysis of representative liver samples from each groups to qualitatively evaluate the liver damage extent.

8. Figure S4. Surface chemistries dependent protein binding pattern might perturb the inflammatory process induced by the ALI plasma.

9. Figure S5. PSi treatment can modulate the immunostimulative effect of ALI plasma through protein corona formation.

10. Figure S6. N-TO, N-TC and N-Un showed lower protein binding capability comparing to TO, TC and Un.

11. Figure S7. Reductive back bonds from PSi NPs had limited effect on modulating the intracellular ROS *in vitro*.

12. Figure S8. Effects of PSi NPs on the cellular viability and cellular morphology of RAW 264.7 cells

13. Figure S8. PSi could consume ROS yet showed limited effect on reversing ROS induced cellular apoptosis under specific condition.

Experimental Section

Fabrication and characterization of different PSi NPs: The PSi particles were produced by electrochemically etching monocrystalline boron–doped *p*+ type Si ⟨100⟩ wafers (Cemat Silicon S.A., Poland) with a resistivity of 0.01–0.02 Ω·cm in a 1:1 (v/v) aqueous hydrofluoric acid (38%)–EtOH electrolyte with a current density of 50 mA/cm^2 in the dark, as described elsewhere.[1] The porous layer was detached from the substrate as a film by increasing the current density to the electro-polishing regime. TO films were made by exposing the fresh films to ambient air for 2 h at 300 °C. Thermally hydrocarbonized PSi (THC) films were firstly obtained by heating at 500 °C under 1:1 N_2-acetylene flow for 15 min. TC films were obtained by heating the THC films absorbed with acetylene at 820 °C for 10 min. Un films were obtained by treating THCPSi films in undecylenic acid at 120 °C for 16 h to obtain terminated carboxylic acid films. The corresponding NPs were produced by wet ball milling the corresponding films. The size sorting and change of the suspension media was achieved by centrifugation of the Un, TC and TO PSi NPs.

The morphology of PSi NPs was observed by transmission electron microscope (TEM, Tecnai 12, FEI Company, USA) at an acceleration voltage of 80 kV. Nanoparticle size and zeta-potential measurement was performed with Zetasizer NanoZS (Malvern Instruments Ltd., UK). The porous properties of the PSi particles were studied using N_2 adsorption/desorption method. The surface areas and pore characteristics of the particles were determined from the obtained data using the Brunauer–Emmet–Teller and Barret–Joyner–Halenda theories. Surface properties of different PSi NPs were investigated by FTIR with a Vertex 70 spectrometer (Bruker Optics, USA), using a horizontal attenuated total reflectance (ATR) accessory (MIRacle, PIKE Technologies, USA).

Animal model and PSi administration: All experiments involving animals were performed in compliance with the guidelines from the Institutional Animal Care and Use Committee at Experimental Animal Centre in Xiamen University, China. Eight- to ten-weeks-old male C57BL/6 mice were randomly allocated to different groups, further administered with APAP (Sigma) at the dosage of 200 mg/kg via oral gavage for inducing ALI. 3 h post ALI model establishment, mice were separately injected *i.v.* with 0.3 or 3 mg/kg of TO, TC and Un, and saline was injected as control. 48 h post PSi administration, all mice were sacrificed and blood samples were collected from heart for further measurement. Dissected liver tissues were directly stored at –80 °C, or fixed in 4% paraformaldehyde, embedded in paraffin, and then processed for tissue-section staining.

Whole blood and serological samples analysis: Whole blood cells analysis was conducted by an automatic biomedical analyzer (VetScan HM5, ABAXIS, USA), according to the manufacturer's instruction. Serum ALT, AST and ALP levels were determined by the corresponding kits. ALT (140118005, Mindray, China), AST (140218004, Mindray, China), ALP (140318005, Mindray, China). Then the prepared samples were measured by an automatic biomedical analyzer (BS-240vet, Mindray, China).

qPCR analysis: For *in vitro* qPCR analysis, RAW 264.7 macrophage cells were treated with different PSi at the concentration of 20 μg/mL. After incubating for 1.5 h, cells were harvested and total RNA was isolated in the presence of Trizol Reagent (Magen, China). cDNA was synthesized using the TransScript One-Step gDNA Removal and cDNA Synthesis SuperMix (Transgen Biotech, China), according to the manufacturer's instructions. Gene expression was detected using a SYBR Green Master Mix (YEASEN, China) and an ABI Step One Plus Real-Time PCR System. Mice primer sequences used in the polymerase chain reaction were shown as followings. TNF-α: 5' - CAG CCT CTT CTC ATT CCT GCT TGT G

- 3', 5' -CTG GAA GAC TCC TCC CAG GTA TAT - 3'; CXCL1: 5' - AGC TTC AGG GTC AAG GCA AG - 3', 5' - CTG CAC CCA AAC CGA AGT - 3'; CCL2(MCP-1): 5' - AGG TGT CCC AAA GAA GCT GTA - 3', 5' - ATG TCT GGA CCC ATT CCT TCT - 3'; IL-6: 5' - TAG TCC TTC CTA CCC CAA TTT C - 3', 5' -TTG GTC CTT AGC CAC TCC TTC - 3'; IL-1β: 5' - GAA ATG CCA CCT TTT GAC AG - 3', 5' - CCA CAG CCA CAA TGA GTG AT- 3'; GAPDH: 5' – GCC TTC CGT GTT CCT ACC C - 3' , 5' – TGC CTG CTT CAC CAC CTT C- 3'.

For *ex vivo* qPCR experiments, 3 h post ALI-establishment, mice were sacrificed and the ALI plasma was collected from heart blood, whereas healthy plasma was collected from healthy mice. DMEM medium containing 10% of ALI plasma or healthy plasma was referred as ALI medium and healthy medium respectively. RAW 264.7 cells were treated with healthy medium, ALI medium with or without 20 μg/mL of different PSi for 1.5 h, then total RNA was isolated and analysed, as described above.

For *in vivo* qPCR, liver samples from each mice were prepared and analysed, as described above.

ELISA assay: For *in vivo* ELISA test, liver samples from each mice were harvested and stored at –80 °C, before the test, all liver samples were prepared in ice-cold saline. IL-1β, IL-6 and TNF-α levels were analyzed in the same supernatants by using the mouse ELISA Kit (cat. EM001, EM004, EM008 ExCell Biotech, China). All the assays were performed according to the manufacturer's instructions. Absorbance was measured at 450 nm with a Varioskan Flash Spectral Scanning Multimode Reader (Thermo Fisher Scientific, USA), and the cytokine quantities were calculated through the use of standard curves for each recombinant cytokine.

For *ex vivo* ELISA test, healthy and ALI medium with or without PSi at the concentration of 20 μg/mL were incubated with RAW 264.7 macrophage cells for 24 h, then the IL-1β, IL-6

and TNF-α levels in blank healthy medium, blank ALI medium and the corresponding cell culturing medium were analyzed, as described above.

For analyzing the specific cytokine binding to each types of PSi NPs, 60 pg/mL of each cytokines solutions were incubated with 2, 10, 20 and 50 μg/mL of different PSi NPs under 4 °C for 24 h, then the NPs were removed via centrifugation and the concentration of each cytokines in the supernatant were measured as described above.

Protein corona analysis: 200 μg of each type of PSi was incubated in 4 mL of 40% AL I mice plasma in DMEM medium at 4 °C for 4 h. After incubation, the NPs were collected by centrifugation and washed twice with Milli-Q water. The proteins were extracted from the plasma treated NPs with SDS-PAGE. The opsonized NPs (40 μg) were suspended in the 4X Loading buffer (62.5 mM Tris-HCL pH = 6.8, 2% SDS (W/V), 10% glycerol, 0.04 M DTT and 0.01% bromophenol blue (W/V)) and incubated at 100 °C for 5 min in order to release and denaturate the adsorbed proteins. The samples were run on a 12% of SDS-PAGE-gel for 2 h at a constant voltage of 100 V. The gel was stained with 0.025% of Coomassie brilliant blue (Beyotiome, China).

Protein corona composition extraction and purification: The proteins were eluted from nanoparticles with 1% of Rapigest (m/v) in 50 mM of ammonium bicarbonate. The elution products were firstly reduced in 20 mM of dithiothreitol (DTT) (Sigma) at 95 °C for 5 min, and subsequently alkylated in 50 mM of iodoacetamide (IAA) (Sigma) for 30 min in the dark at room temperature (RT). After alkylation, the samples were centrifugated at 20000g for 10 min, the supernatant were transferred to a 3 kDa centrifugal spin filter (Millipore) and sequentially washed with 200 μL of 8 M of urea for three times and 200 μL of 50 mM of ammonium bicarbonate for two times by centrifugation at 13500g. Next, tryptic digestion was performed by adding trypsin (Promega) and Lys-C (Wako) at 1:50 (enzyme/substrate, m/m) in

200 μL of 50 mM of ammonium bicarbonate at 37 ºC for 16 h. Peptides were recovered by transferring the filter to a new collection tube and spinning at 13500g. To increase the yield of peptides, the filter was washed twice with 100 μL of 50 mM of ammonium bicarbonate. Peptides were desalted by StageTips and consequently lyophilized followed by labeling with TMT10plex (Pierce), according to the manufacturer's instruction.

100 μg of labeled peptides were off-line fractionated by bRP using a Waters XBridge BEH C18 5 μm 4.6 × 250 mm column (Waters) on an Ultimate 3000 high-pressure liquid chromatography (HPLC) system (Dionex) operating at 1 mL/min. Buffer A (5 mM of ammonium formate pH 10) and buffer B (5 mM of ammonium formate pH 10, 90% (v/v) ACN) were adjusted to pH 10 with ammonium hydroxide. Peptides were separated by a linear gradient from 5% B to 40% B in 54 min followed by a linear increase to 70% B in 6 min. A total of 60 fractions were collected. The 60 fractions were concatenated to 20 fractions, all the peptide fractions were lyophilized to dryness.

LC-MS/MS analysis: All MS experiments were performed on a nanoscale EASY-nLC 1200UHPLC system (Thermo Fisher Scientific) connected to an Orbitrap Fusion Lumos equipped with a nanoelectrospray source (Thermo Fisher Scientific). Mobile phase A contained 0.1 % of formic acid (v/v) in water; mobile phase B contained 0.1% of formic acid in 80% of acetonitrile (ACN). The peptides were dissolved in 0.1% of formic acid (FA) with 2% of acetonitrile and separated on a RP-HPLC analytical column (75 μm × 25 cm) packed with 2 μm C18 beads (Thermo Fisher Scientific) using a linear gradient ranging from 9% to 32% of ACN in 90 min and followed by a linear increase to 50% B in 20 min at a flow rate of 300 nL/min. The Orbitrap Fusion Lumos acquired data in a data-dependent manner alternating between full-scan MS and MS^2 scans. The spray voltage was set at 2.2 kV and the temperature of ion transfer capillary was 300 °C. The MS spectra (350−1500 m/z) were collected with 60000 resolution, AGC of 4×10^5, and 50 ms maximal injection time. Selected

ions were sequentially fragmented in a 3 s cycle by HCD with 38 % normalized collision energy, specified isolated windows 0.7 m/z, 30000 resolution. AGC of 5×10^4 and 80 ms maximal injection time were used. Dynamic exclusion was set to 40 s. Unassigned ions or those with a charge of 1+ and >7+ were rejected for MS/MS.

Mass spectrometry data analysis: Raw data was processed using Proteome Discoverer (PD, version 2.2), and MS/MS spectra were searched against the reviewed SwissProt human proteome database. All searches were carried out with precursor mass tolerance of 20 ppm, fragment mass tolerance of 0.02 Da, oxidation (Met) (+15.9949 Da)，TMT6plex（Lys）(229.163 Da) and acetylation (protein N-terminus) (+42.0106 Da) as variable modifications, carbamidomethylation (Cys) (+57.0215 Da), TMT6plex (N-terminal) (229.163Da) as fixed modification and three trypsin missed cleavages allowed. Only peptides with at least six amino acids in length were considered. The peptide and protein identifications were filtered by PD to control the false discovery rate (FDR) <1%. At least one unique peptide was required for protein identification.

R-code:

```
library(gplots)
library(RColorBrewer)
options(stringsAsFactors = F)
datExp = read.table('ExpressionDataForDEG.txt',sep='\t',header=T)
View(head(datExp))
datExp1 = datExp[, -1]
rownames(datExp1) = datExp[, 1]
View(datExp1)
datExp1 = as.matrix(datExp1)
```

```
heatmap.2(datExp1,col = greenred(75),
          hclust=function(x) hclust(x,method = 'ward.D2'),
          distfun=function(x) dist(x,method='euclidean'),
          scale = "row",dendrogram = 'both',
          key = TRUE, symkey = FALSE, density.info = "none",
          trace = "none", cexRow = 1,
```

PSi degradation study: To evaluate *in vitro* PSi degradation kinetics, 150 µg of different PSi were incubated in 3 mL of 1× phosphate-buffered saline buffer (PBS, 6.7 mM, pH 7.4) containing 1% of Poloxamer 188 (P188) with or without 2 mM of 3-morpholinosydnonimine (SIN-1, Enzo Life Sciences, USA), under orbital agitation of 100 rpm at 37 °C. The pH values of the solution were constantly monitored during the experiments. At designated time intervals, aliquots were sampled and replaced with fresh corresponding degradation buffer. The resulting liquid was stored at 4 °C for later analysis of total silicon by microwave plasma atomic emission spectroscopy (MP-AES, Varian Inc. Santa Clara, CA, USA).

Flow cytometry analysis: Inflammation was simulated by activating RAW 264.7 cells with LPS and interferon γ (IFN-γ), to avoid false positive results induced by LPS, IFN-γ and DCFH-DA absorption to NPs, prior to PSi administration, cells were thoroughly washed to remove free LPS, IFN-γ and DCFH-DA. RAW 264.7 macrophage cells were separately seeded in 12-well plates at the concentration of 5×10^5 cells per well. Following cell attachment to the wells, the cells were incubated with or without 10 pg/mL of IFN-γ and 1 µg/mL LPS for 4 h, then cells were washed with 1× PBS (6.7 mM, pH 7.4) and then 0.5 mL of 10 µM DCFH-DA was added. After 1 h, the DCFH-DA containing medium was removed and cells were washed with 1× PBS for 3 times followed by adding 2, 10, 20 and 50 µg/mL of each PSi. After 24 h, the cells were harvested and fixed with 4% of paraffinaldehyde in 1×

PBS for 15 min at room temperature, then the cells were washed with 1×PBS for 3 times and kept under 4 °C for further measurement. At least 5000 events were collected on a LSR II flow cytometer (BD Biosciences, USA) with a laser excitation wavelength of 488 nm using FACS Diva software.

Statistical analysis: One-way ANOVA analysis and two-tailed Students' *t*-test was used to compare values among different experimental groups using Excel 2010 and Graphpad Prism 5 respectively. $P < 0.05$ was considered a statistically significant change. *$p < 0.05$; **$p < 0.01$; ***$p < 0.0005$; ****$p < 0.0001$; ns, not significant. All the values were presented as mean ± standard deviation (SD) of at least triplicate experiments.

Table S1 Quantitative characterization of different PSi NPs.

	TO	TC	Un
Size (nm)	175 ± 15	154 ± 5	180 ± 6
ζ-potential (mV)	-26 ± 2	-25 ± 5	-30 ± 1
Specific surface area (m^2/g)	203 ± 11	212 ± 4	242 ± 1
Total pore volume (cm^{-3}/g)	0.57 ± 0.04	0.52 ± 0.07	0.73 ± 0.01
Pore diameter (nm)	11.3 ± 0.2	9.9 ± 1.4	12.0 ± 0.1
Water contact angle (°)	51 ± 5	62 ± 4	122 ± 6

Table S2 Quantitative characterization of different new PSi NPs.

	N-TO	N-TC	N-Un
Specific surface area (m^2/g)	102 ± 4	136 ± 4	112 ± 9
Total pore volume (cm^{-3}/g)	0.12 ± 0.01	0.15 ± 0.01	0.10 ± 0.02
Pore diameter (nm)	4.7 ± 0.1	4.4 ± 0.1	3.7 ± 0.3

Table S3. List of abbreviations and variations.

Abbreviation	Description
PSi NPs	Porous silicon nanoparticles
ALI	Acute liver inflammation
TO	Thermal oxidized PSi NPs
TC	Thermal carbonized PSi NPs
Un	Undecylenic acid modified thermal hydrocarbonized PSi NPs
WCA	Water contact angle
TEM	Transmission electron microscopy
DLS	Dynamic light scattering
FTIR	Fourier transform infrared spectroscopy
APAP	Acetaminophen
TO/TC/Un L	TO/TC/Un at low concentration (0.3 mg/kg)
TO/TC/Un H	TO/TC/Un at high concentration (3 mg/kg)
WBC	White blood cells
GRA	Granulocytes
MON	Monocytes
LYM	Lymphocytes
H&E	Hematoxylin and eosin
TUNEL	TdT-mediated dUTP Nick-End labeling
AST	Aspartate aminotransferase
ALT	Alanine aminotransferase
ALP	Alkaline phosphatase
GSH	Glutathione
qPCR	Quantitative real-time polymerase chain reaction
IL-1β	Interleukin-1β
IL-6	Interleukin-6
TNF-α	Tumor necrosis factor α

CXCL-1	chemokine (C-X-C motif) ligand 1
CCL-2	chemokine (C-C motif) ligand 2
ELISA	Enzyme linked immunosorbent assay
SDS-PAGE	Sodium dodecyl sulphate-polyacrylamide gel electrophoresis
TMT	Isobaric Tags for Tandem Mass Tag
DAVID	Database for Annotation, Visualization and Integrated Discovery
GO	Gene ontology analysis
CC	Cellular component
BP	Biological process
MF	Molecular function
FDR	False discovery rate
DAMP	Damage-associated molecular patterns
NF-κB	Nuclear factor κB
IκBα	Inhibitor κ-Bα
ALI medium	DMEM medium containing 10% mice plasma collected from ALI mice
$ALI^{TO+/TC+/Un+}$	RAW 264.7 macrophage cells separately treated with ALI medium containing 20 μg/mL of TO/TC/Un for 24 h
$ALI^{TO-/TC-/Un-}$	ALI medium pre-incubated with 20 μg/mL of TO/TC/Un for 4 h, followed by removing the NPs, the resided medium was incubated with RAW 264.7 macrophage cells for 24 h
TO/TC/Un-corona+	FBS free DMEM medium containing 20 μg/mL TO/TC/Un recovered from ALI medium treatment
TO/TC/Un-corona-	FBS free DMEM medium containing 20 μg/mL pristine TO/TC/Un
N-TO/N-TC/N-Un	New batch of TO/TC/Un with significantly lower porosity
$ALI^{NTO+/NTC+/NUn+}$	RAW 264.7 macrophage cells separately treated with ALI medium containing 20 μg/mL of N-TO/N-TC/N-Un for 24 h
$ALI^{NTO-/NTC-/NUn-}$	ALI medium pre-incubated with 20 μg/mL of N-TO/N-TC/N-Un for 4 h, followed by removing the NPs, the resided medium was incubated with RAW 264.7 macrophage cells for 24 h
DCFH-DA	2',7'-dichlorodihydrofluorescein diacetate

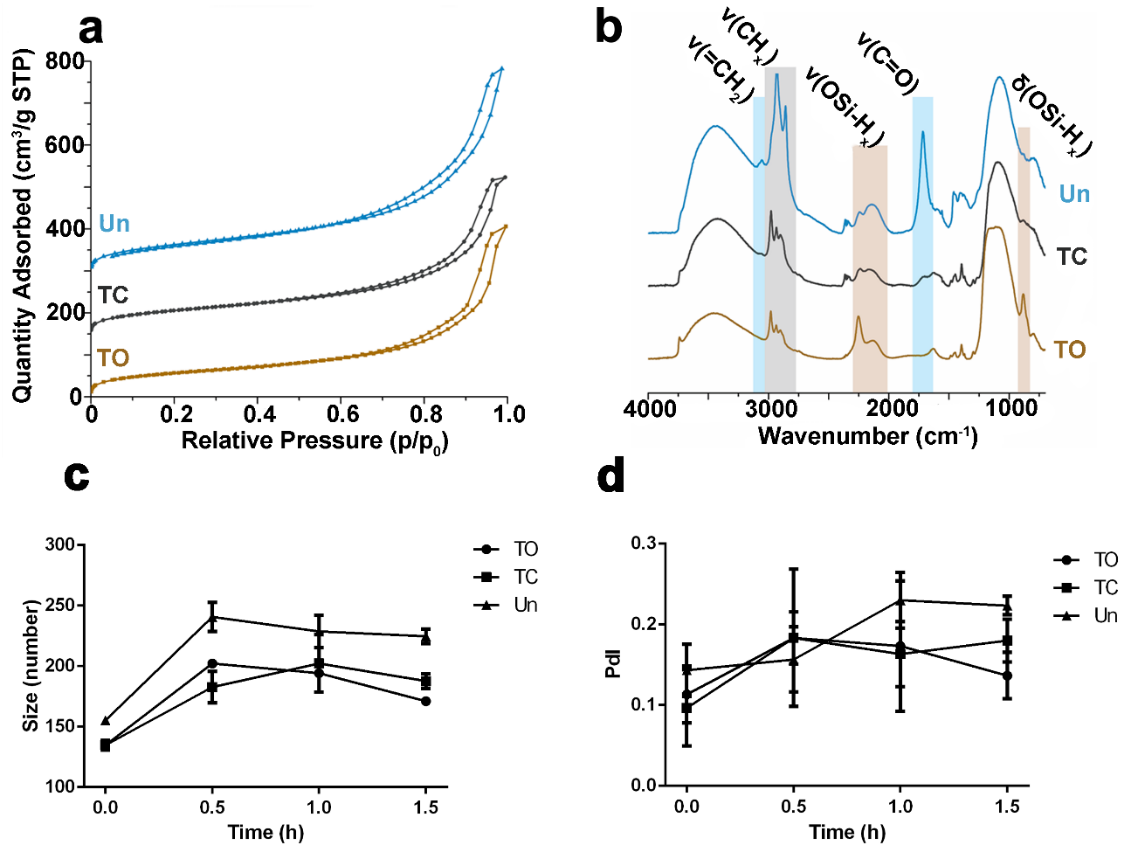

Figure S1. Characterization of different PSi NPs. (a) N_2 adsorption–desorption isotherms of different PSi NPs, confirming the overall similar porosity of these NPs. (b) FTIR absorbance spectra of different PSi NPs and the corresponding characteristic bands confirming the altered surface chemistries. The most distinguished peaks from TO could be found at 3740 cm^{-1} and 882 cm^{-1}, which were respectively attributed to Si-OH and -OySi-Hx. Whereas the typical ν (C=O) band at 1715 cm^{-1} from Un confirmed the successful undecylenic acid hydrosilylation, and this peak was also visible from TC, which was due to the acetylene treatment and the following high temperature annealing. Different hydrophobicity from different PSi NPs was observed from the water related bands at 1630 cm^{-1}, which were more prominent from TO and TC but not Un. It should be noted that, despite different surface stabilization methods, there were still some hydrides (-OySi-Hx) remains as can be confirmed by the PSi hydride stretch region in 2100–2300 cm^{-1}. (c) The size and (d) PdI changes of each type of PSi NPs after incubating with 50% human blood plasma under 37°C, confirming PSi NPs were relatively stable under pathological condition.

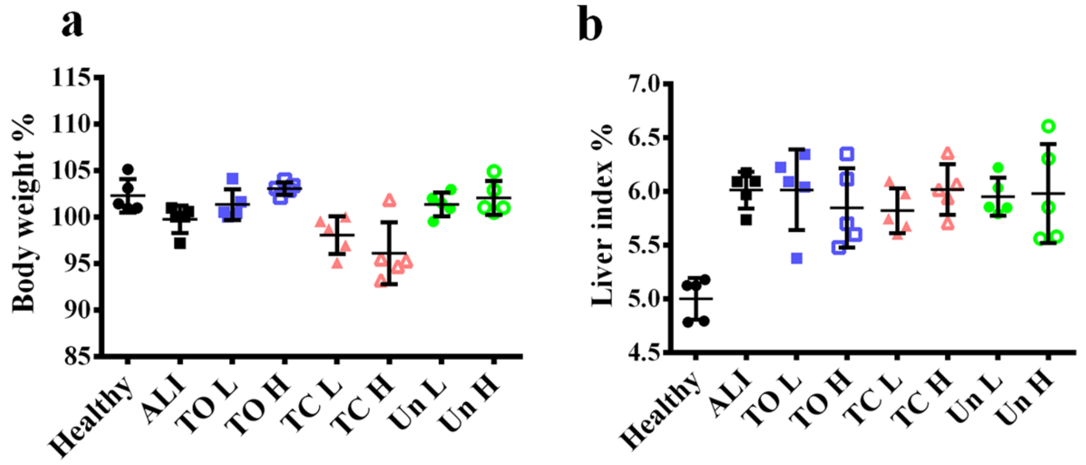

Figure S2. Body weight and liver index of mice from each group. (a) The body weight of mice was separately recorded before ALI establishment and after 48 h of PSi administration. The weights were recorded as percentage of initial bodyweight. Comparing to ALI group, sequential administration of PSi NPs did not affect the bodyweight. (b) 48 h post PSi NPs administration, all mice were sacrificed and the liver were immediately collected for weighting. Comparing to ALI group, TO, TC and Un administration showed no effect on liver index. Data were shown as mean ± SD (n =5).

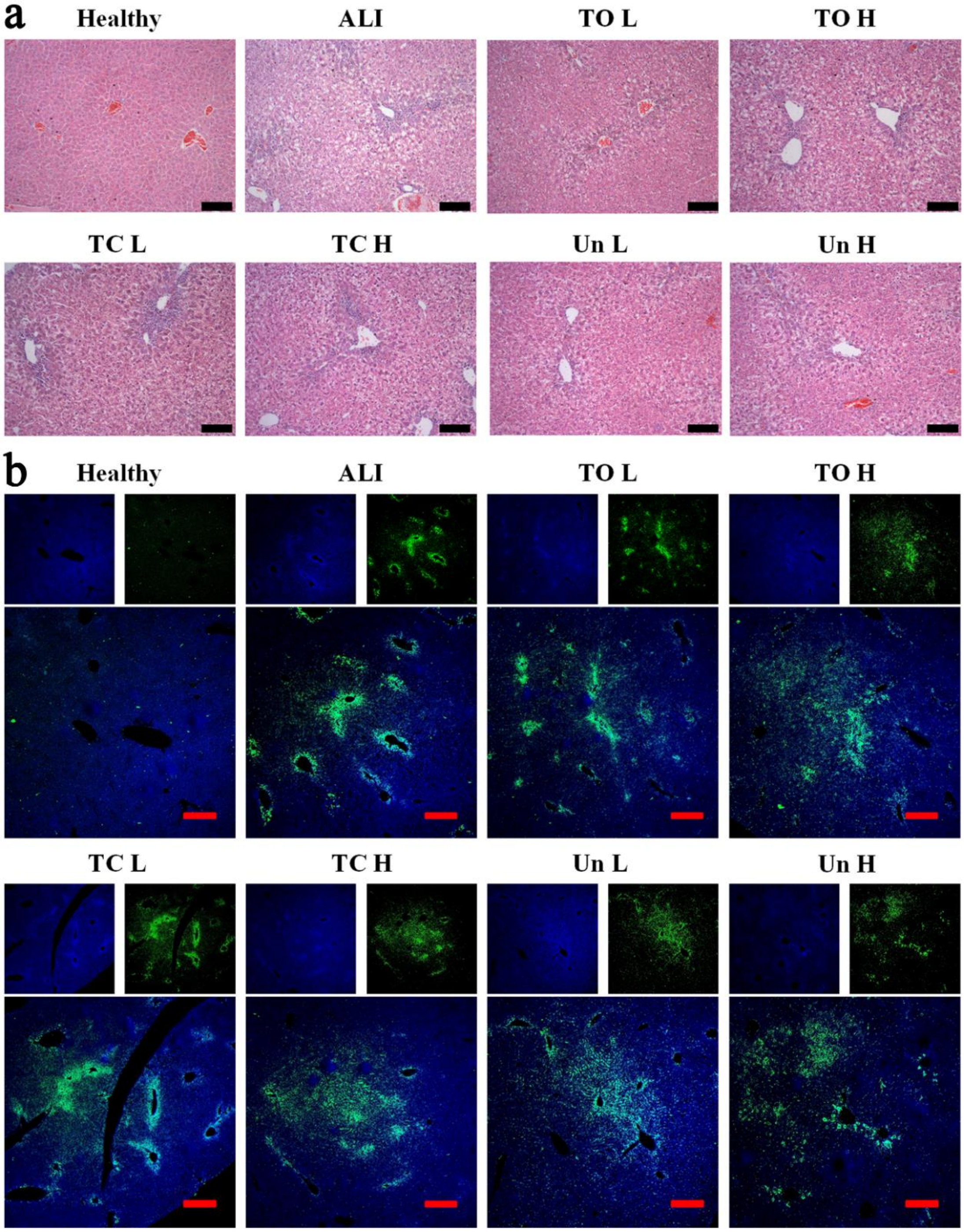

Figure S3. Histological and immunohistological analysis of representative liver samples from each group to qualitatively evaluate the liver damage extent. (a) Hematoxylin and eosin (H&E) staining of representative liver samples from each groups. Piecemeal necrosis, which is featured with a lymphocytic infiltrate into the adjacent parenchyma, and with destruction of individual hepatocytes along the edges of the portal tract, can be observed. All scale bars are 100 μm. (b) TdT-mediated dUTP Nick-End labeling (TUNEL) staining of representative liver samples from each groups. All scale bars are 200 μm.

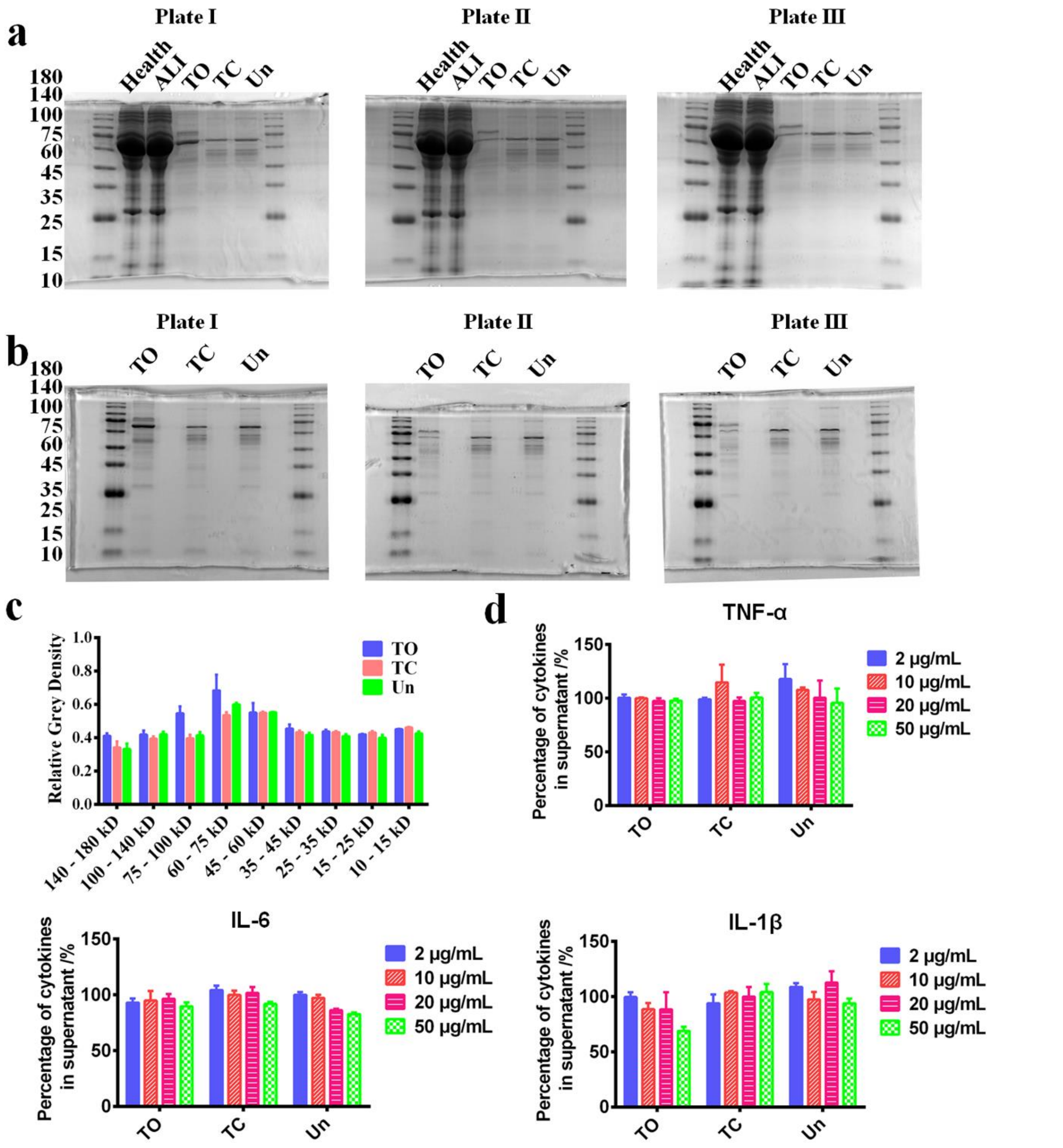

Figure S4. Surface chemistries dependent protein binding pattern might perturb the inflammatory process induced by the ALI plasma. (a-b) Uncropped blots with size marker indications (KDa). SDS-PAGE analysis of plasma protein obtained from PSi NPs-corona complexes, with (a) or without (b) blank healthy plasma and ALI plasma as contrast. (c) Relative amounts of the absorbed proteins within each protein band zone as calculated by the grey-scale value from the gels shown in a, b. Values are expressed as the means ± SD of triplicates. (d) The binding efficiency of three major pro-inflammatory cytokines (TNF-α,

IL-6 and IL-1β) to different PSi NPs. 100 pg/mL standard solutions of different cytokines were incubated with 2, 10, 20 or 50 μg/mL of each type of PSi NPs for 24 h at 4 °C, the amount of each cytokines within the supernatant were further evaluated via ELISA method.

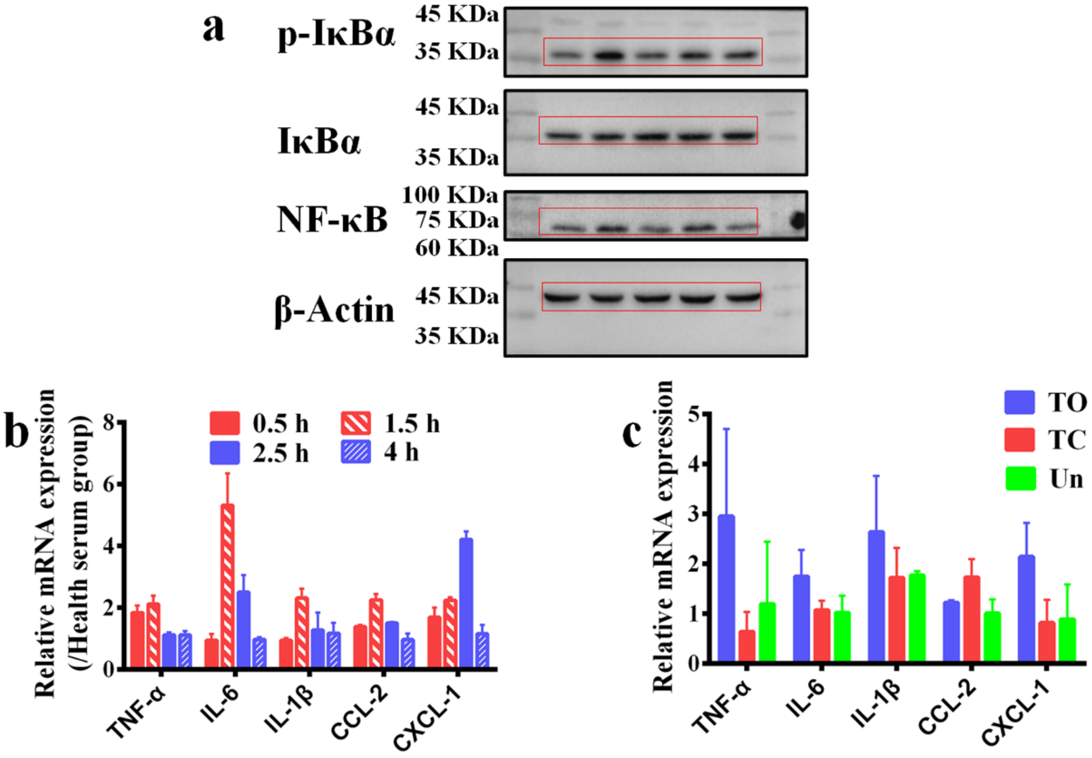

Figure S5. PSi treatment can modulate the immunostimulative effect of ALI plasma through protein corona formation. (a) Uncropped blots for NF-κB signaling pathway with size marker indications. (b) ALI medium could provoke a pro-inflammatory cascade by enhancing the pro-inflammatory cytokines/chemokines mRNA expression in a time dependent manner. The mRNA expression levels of each cytokines/chemokines from cells treated with healthy medium at the corresponding time-point was fixed as 1. Values are expressed as the means ± SD of triplicates. (c) RAW cells were treated with 20 μg mL^{-1} of PSi NPs dispersed in DMEM medium containing 10% of FBS for 1.5 h, then cells were harvested and the mRNA expression level of each cytokines/chemokines were determined via qPCR method. The mRNA expression levels of each cytokines/chemokines from cells treated with blank DMEM medium containing 10% of FBS was fixed as 1. Values are expressed as the means ± SD of triplicates.

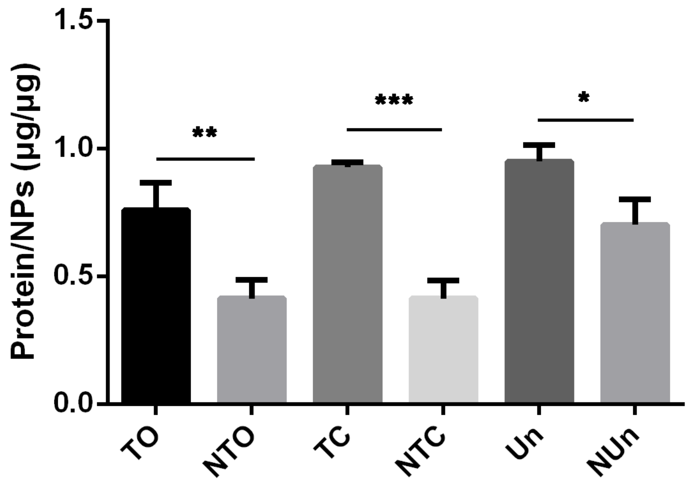

Figure S6. N-TO, N-TC and N-Un showed lower protein binding capability comparing to TO, TC and Un. Values are expressed as the means ± SD of triplicates.

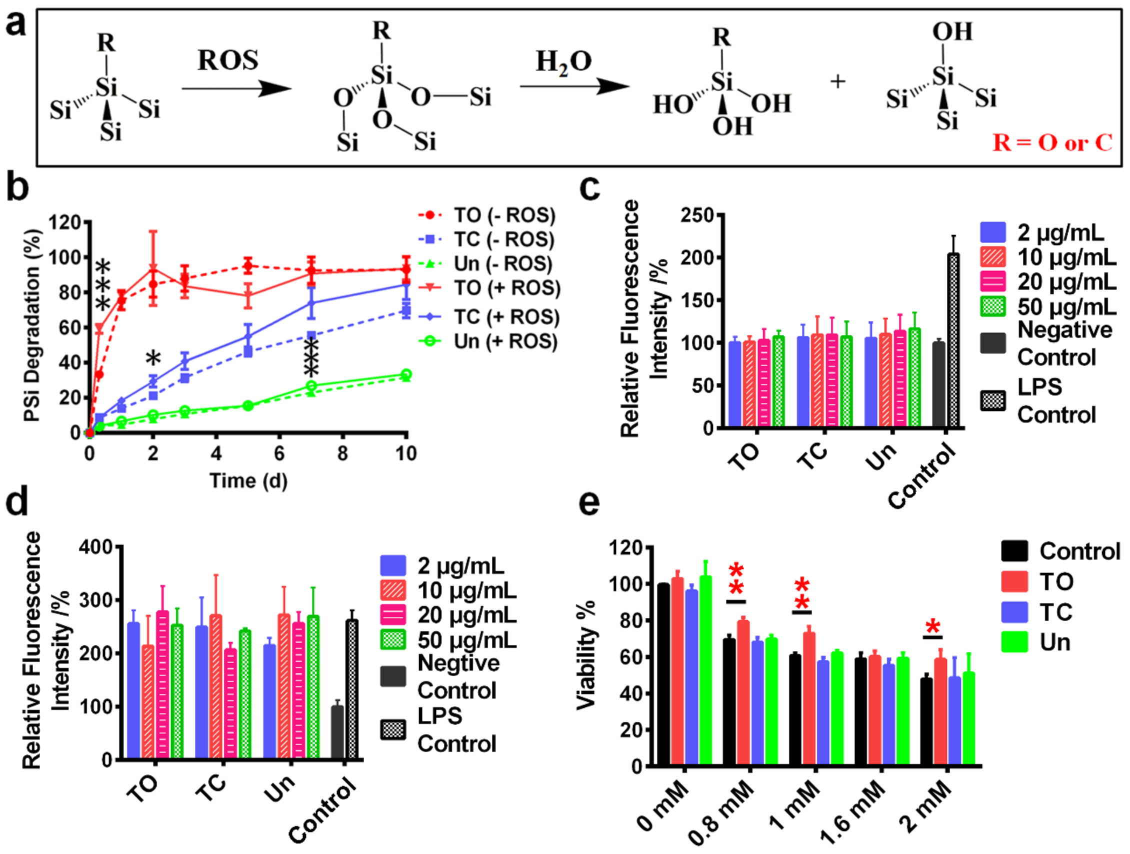

Figure S7. Reductive back bonds from PSi NPs had limited effect on modulating the intracellular ROS *in vitro*. (a) Schematic illustration of ROS facilitating PSi NPs degradation. (b) Addition of extra ROS accelerated the PSi NPs degradation in different extent. Different PSi NPs at the concentration of 50 μg/mL were dispersed in 1× PBS (pH 7.4) containing 1% of Poloxamer 188 under 37 °C with orbital agitation. ROS generated by adding 2 mM of SIN-1. (c–d) PSi-mediated intracellular ROS level under a normal or inflammatory condition. Blank RAW 264.7 macrophages cells (c) or cells pre-treated with 1 μg/mL of LPS and 10 pg/mL of IFN-γ for 4 h (d) were incubated with 2, 10, 20 or 50 μg/mL of each type of PSi NPs for 24 h. Then the intracellular ROS levels were measured by a DCFH-DA method using flow cytometry. Blank cells or cells pre-treated with 1 μg/mL of LPS and 10 pg/mL of IFN-γ for 4 h were separately set as negative control or LPS control. **(e)** TO PSi NPs attenuated the ROS induced cellular apoptosis under a specific condition. 0, 0.8 1, 1.6 or 2 mM of H_2O_2 containing medium was incubated with 50 μg/mL of different PSi for 24 h, further applied to incubate HepG2 cells. Cellular viability of each group analyzed via an

ATP-luminescence based method. Values are expressed as the means ± SDs of triplicates. Statistical significance was assessed by two-tailed Student's *t*-test; *$p < 0.05$, **$p < 0.01$ and ***$p < 0.0005$.

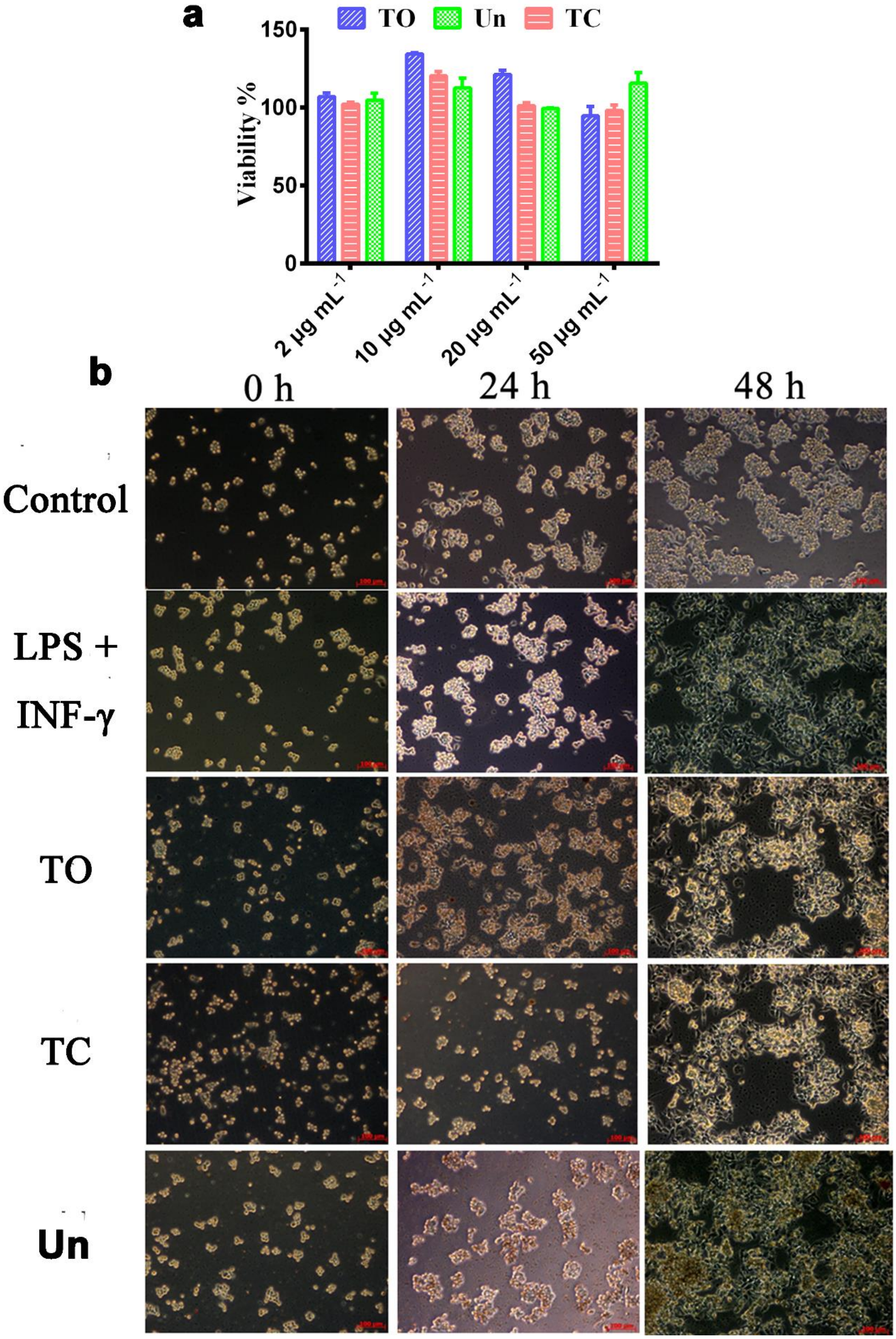

Figure S8. Effects of PSi NPs on the cellular viability and cellular morphology of RAW 264.7 cells (a) The viability of RAW 264.7 macrophages after incubating with TO, TC and Un at different concentrations for 24 h. Values are expressed as the means ± SDs of triplicates.

(b) The actual RAW 264.7 macrophages images after stimulating with LPS and IFN-γ and further incubating with TO, TC and Un at the concentration of 50 μg mL^{-1} for 24 and 48 h. All scale bars represent 100 μm.

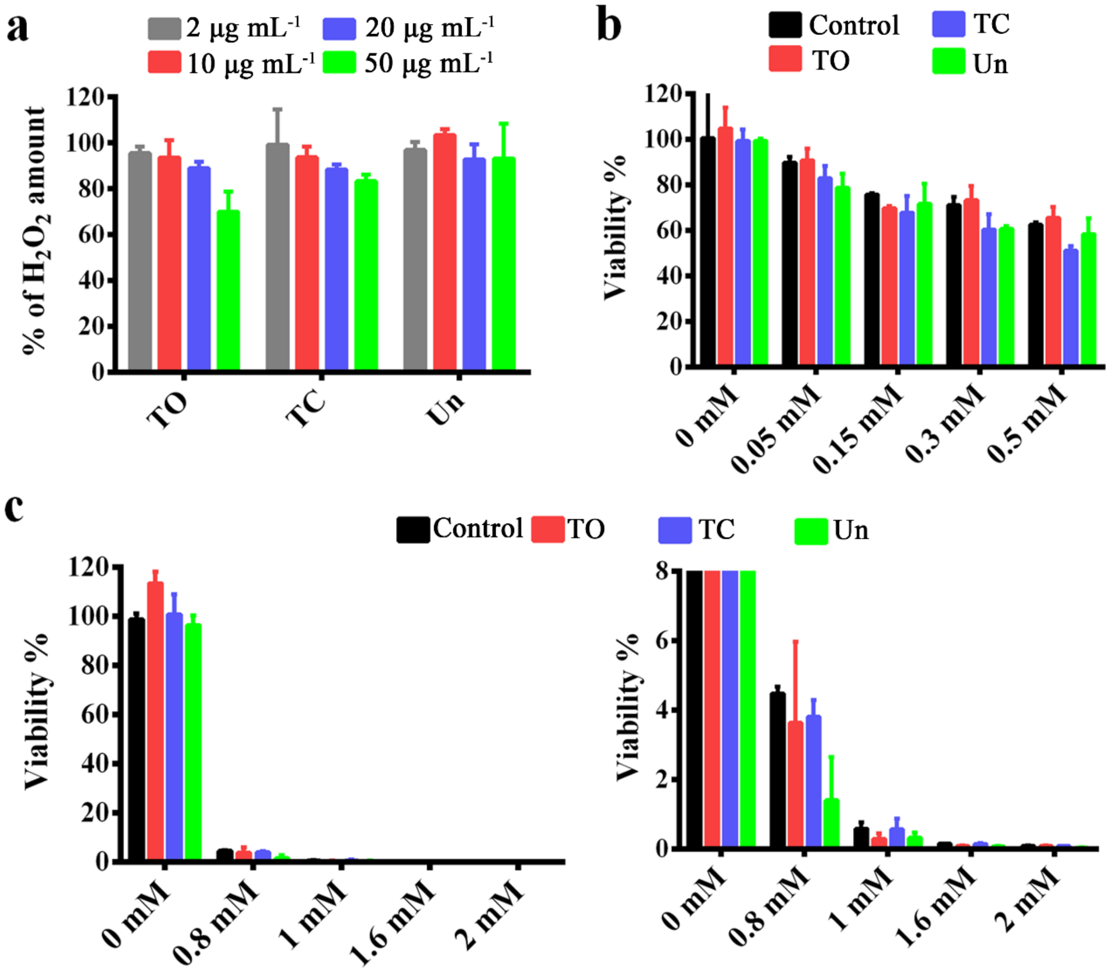

Figure S9. PSi could consume ROS yet showed limited effect on reversing ROS induced cellular apoptosis under specific condition. (a) Co-incubating PSi with H_2O_2 for overnight could reduce the H_2O_2 amount in the supernatant. TO, TC and Un at different concentration were incubated 1 mM of H_2O_2 for 24 h, then the H_2O_2 concentration was determined via DCFH-DA method. (b-c) Simultaneous co-administration of PSi NPs and H_2O_2 containing medium to HepG2 cells can not reverse H_2O_2 induced cellular apoptosis. Data were shown as mean ± SD (n = 3).

References

[1] J. Salonen, E. Laine, L. Niinistö, J. Appl. Phys. 2002, 91, 456; Z. Liu, Y. Li, W. Li, C. Xiao, D. Liu, C. Dong, M. Zhang, E. Mäkilä, M. Kemell, J. Salonen, Adv. Mater. 2018, 30, 1703393; J. Salonen, V. P. Lehto, E. Laine, Appl. Phys. Lett. 1997, 70, 637.
